# Mal3 is a multi-copy suppressor of the sensitivity to microtubule-depolymerizing drugs and chromosome mis-segregation in a fission yeast *pka1* mutant

**DOI:** 10.1371/journal.pone.0214803

**Published:** 2019-04-11

**Authors:** Takuma Tanabe, Masayuki Yamaga, Makoto Kawamukai, Yasuhiro Matsuo

**Affiliations:** 1 Department of Life Sciences, Faculty of Life and Environmental Science, Shimane University, Matsue, Japan; 2 Institute of Agricultural and Life Sciences, Academic Assembly, Shimane University, Matsue, Japan; University of Illinois at Chicago, UNITED STATES

## Abstract

The cAMP-dependent protein kinase Pka1 is known as a regulator of glycogenesis, transition into meiosis, chronological aging, and stress responses in the fission yeast, *Schizosaccharomyces pombe*. We demonstrated here that Pka1 is responsible for normal growth in the presence of the microtubule-destabilization drug TBZ and proper chromosome segregation. The deletion of the *pka1* gene resulted in the TBZ-sensitive phenotype and chromosome mis-segregation. We isolated the *mal3* gene as a multi-copy suppressor of the TBZ-sensitive phenotype in the *pka1Δ* strains. Overexpression of the CH domain (1–143) or the high-affinity microtubule binding mutant (1–143 Q89R) of Mal3 rescued the TBZ-sensitive phenotype in the *pka1Δ* and *mal3Δ* strains, while the EB1 domain (135–308) and the mutants defective in microtubule binding (1–143 Q89E) failed to do so in the same strains. Chromosome mis-segregation caused by TBZ in the *pka1Δ* or *mal3Δ* strains was suppressed by the overexpression of the Mal3 CH domain (1–143), Mal3 CH domain with the coiled-coil domain (1–197), or full-length Mal3. Overexpression of EB1 orthologs from *Saccharomyces cerevisiae*, *Arabidopsis thaliana*, *Mus musculus*, or *Homo sapiens* suppressed the TBZ-sensitive phenotype in the *pka1Δ* strains, indicating their conserved functions. These findings suggest that Pka1 and the microtubule binding of the Mal3 CH domain play a role in the maintenance of proper chromosome segregation.

## Introduction

Microtubules are the major cytoskeletal fibers in eukaryotic cells, which maintain the structure of cells and provide platforms for substance transport. Microtubules grow by the polymerization of the tubulin dimer, which consists of the α-tubulin and ß-tubulin, and shrink by depolymerization. In mitosis, microtubules dramatically change their structure and regulate chromosome segregation. Microtubule associated proteins (MAPs), such as XMAP215, EB1, CLIP170, and CLASP1, regulate microtubule function and stability [1, 2]. Microtubule destabilizing drugs such as nocodazole, benomyl, thiabendazole (TBZ), and carbendazim (MBC), inhibit microtubule polymerization, especially that of ß-tubulin, resulting in shorter microtubules. These microtubule destabilizing drugs have benzimidazole as a basal structure and are used as anti-fungal and anti-helminthic drugs [3, 4]. Because TBZ and MBC inhibit microtubules formation and cause chromosome mis-segregation, these drugs have been used to understand the mechanism of microtubule formation and chromosome segregation [5, 6].

EB1 was first isolated as a protein that interacts with the C-terminus of adeno tumor polyposis coli, by screening in a yeast two-hybrid system [7]. EB1 is one of the MAPs that bind to microtubules, especially at the microtubules plus-end, and therefore, is known as a plus-end tracking protein (+TIP) [8], which also include EB1, CLIP-170, XMAP215, and CLASP1 [1]. The +TIP proteins play a role in the proper formation of microtubules, cell polarity, and cell elongation [1]. EB1 has the Calponin homology (CH) domain at the N-terminus, the coiled-coil domain in the middle, and the end-binding (EB1) domain at the C-terminus [9]. The CH and the coiled-coil domains are responsible for the dimerization of EB1 [10]. The EB1 domain plays a role in the interaction with binding partners such as EB1, CLIP-170 and CLASP1 [2].

Mal3, in the fission yeast *Schizosaccharomyces pombe*, is a homolog of EB1, which is required for microtubule integrity and the maintenance of cell morphology [11]. The deletion of *mal3* gene results in shorter microtubules increases the loss of mini-chromosome during chromosome segregation, and exhibits the TBZ-sensitive phenotype [11]. Mal3 mutants have been analyzed for their activity on microtubule binding by several groups. Mal3 (K63D) and Mal3 (Q89E) fail to bind to microtubules, while Mal3 (Q89A) and Mal3 (Q89R) bind excessively to microtubules, both *in vitro* and *in vivo* [12, 13]. Mal3 (Q89R) causes hyper phosphorylation on the coiled-coil region of Mal3 [12]. However, the kinase responsible for the phosphorylation of Mal3 has not been identified.

The cyclic adenosine monophosphate (cAMP)-dependent protein kinase, also known as protein kinase A (PKA), is a serine/threonine kinase that is highly conserved among organisms ranging from yeasts to mammals [14–17]. cAMP, generated by the adenylate cyclase (Cyr1), from adenosine triphosphate (ATP), binds with Cgs1, a regulatory subunit of PKA, to release and activate Pka1 [18–20]. In *S*. *pombe*, this pathway is known to be involved in glucose repression, chronological aging, regulation of transit from mitosis to meiosis, and stress responses to KCl and CaCl_2_ [14, 21–25]. However, the biological roles of the cAMP/PKA pathway are not fully understood.

In this study, we found that *pka1Δ* showed the TBZ-sensitive and chromosome mis-segregated phenotypes. We screened multi-copy suppressors from a *S*. *pombe* cDNA library that suppress the TBZ-sensitive phenotype of the *pka1Δ* strains and isolated the *mal3* gene. Domain analysis revealed that the CH domain of Mal3 is sufficient for the multi-copy suppression of the TBZ-sensitive and chromosome mis-segregated phenotypes in the *pka1Δ* and *mal3Δ* strains. We show here the novel role of Pka1 in microtubule organization and proposed a novel function for the Mal3 CH domain.

## Materials and methods

### Yeast strains, media, and genetic methods

The *S*. *pombe* strains used in this study are listed in Table 1. Standard yeast culture media and genetic methods were utilized [26, 27]. *S*. *pombe* cultures were grown in either YES medium (0.5% yeast extract, 3% glucose, 225 mg/L adenine, 225 mg/L uracil, 225 mg/L leucine, 225 mg/L histidine, and 225 mg/L lysine) or synthetic minimal medium (EMM) with appropriate auxotrophic supplements [27].

**Table 1:** *S*. *pombe* strains used in this study.

Strain	Genotype	Source
PR109	*h*^*-*^ *leu1-32 ura4-D18*	P. Russell
YMP40	*h*^*-*^ *leu1-32 ura4-D18 cgs1*::*ura4*	[22]
YMP36	*h*^*-*^ *leu1-32 ura4-D18 pka1*::*ura4*	[22]
YMP28	*h*^*-*^ *leu1-32 ura4-D18 cyr1*::*ura4*	[22]
YMP58	*h*^*-*^ *leu1-32 ura4-D18 cyr1*::*LEU2 cgs1*::*ura4*	[22]
TTP1	*h*^*-*^ *leu1-32 ura4-D18 mal3*::*natMX6*	This study
TTP2	*h*^*+*^ *leu1-32 ura4-D18 mal3*::*natMX6*	[30]
TTP5	*h*^*-*^ *leu1-32 ura4-D18 cgs1*::*ura4 mal3*::*natMX6*	This study
TTP7	*h*^*-*^ *leu1-32 ura4-D18 pka1*::*ura4 mal3*::*natMX6*	This study
TTP3	*h*^*-*^ *leu1-32 ura4-D18 mal3-GFP(S65T)-natMX6*	[30]
TTP26	*h*^*-*^ *leu1-32 ura4-D18 cgs1*::*ura4 mal3-GFP(S65T)-natMX6*	This study
TTP20	*h*^*-*^ *leu1-32 ura4-D18 pka1*::*ura4 mal3-GFP(S65T)-natMX6*	This study
TTP4	*h*^*-*^ *leu1-32 ura4-D18 mal3-13Myc-natMX6*	This study
TTP24	*h*^*-*^ *leu1-32 ura4-D18 cgs1*::*ura4 mal3-13Myc-natMX6*	This study
TTP22	*h*^*-*^ *leu1-32 ura4-D18 pka1*::*ura4 mal3-13Myc-natMX6*	This study
MY273 (FY8144)	*h*^*-*^ *his2 ade6-M210 Ch16*	NBRP
TTP101	*h*^*-*^ *ade6-M210 leu1-32 ura4-D18 Ch16*	This study
TTP104	*h*^*-*^ *ade6-M210 leu1-32 ura4-D18 pka1*::*ura4 Ch16*	This study
TTP69	*h*^*+*^ *ade6-M210 leu1-32 ura4-D18 Ch16*	This study
TTP70	*h*^*+*^ *ade6-210 leu1-32 ura4-D18 mal3*::*kanMX6 Ch16*	This study
TTP103	*h*^*+*^ *leu1-32 ura4-D18 sad1-mRFP-hphMX6*	This study
TTP110	*h*^*+*^ *leu1-32 ura4-D18 pka1*::*ura4 sad1-mRFP-hphMX6*	This study
TTP113	*h*^*+*^ *leu1-32 ura4-D18 mal3*::*naMX6 sad1-mRFP-hphMX6*	This study
TTP76	*h- leu1-32 ura4-D18 kanMX6-nmt81-GFP-atb2 sad1-mRFP-natMX6*	This study
TTP218	*h- leu1-32 ura4-D18 kanMX6-nmt81-GFP-atb2 sad1-mRFP-natMX6 pka1*::*ura4*	This study

### Construction of *sad1-mRFP* and *mal3-13Myc*

The *sad1-mRFP-*tagged strain was constructed using the recombinant PCR approach described by Krawchuk et al. [28]. The resulting DNA fragments carried the mRFP-natMX6 cassette in the region 3′-downstream of the *sad1* gene. For this purpose, the following oligonucleotides were used: sad1-TAGW (5’-GACGATTGGAAGTTCTTCATC-3’), sad1-TAGX (5’-GGGGATCCGTCGACCTGCAGCGTACGAAGATGAATCTTGACCCGTATTC-3’), sad1-TAGY (5’-GTTTAAACGAGCTCGAATTCATCGATCATCTGCTTCCTCGAGCATC-3’), and sad1-TAGZ (5’-TAAAGAACGAGCGGATGGCTG-3’) for the *sad1-mRFP-natMX6* cassette or *sad1-mRFP-hphMX6* cassette. The PR109 strain was transformed with the resulting *sad1-mRFP-natMX6* cassette or *sad1-mRFP-hphMX6* cassette and the transformants were isolated on YES medium containing clonNAT (nourseothricin) or hygromycin B, respectively. Transformants carrying the mRFP-tagged *sad1* gene were verified by colony PCR [29]. The *mal3-13Myc-*tagged strain was constructed with using the same method as that used in the construction of the *mal3*-GFP strain, as previously described [30].

The other strains (TTP5: *cgs1Δ mal3Δ*, TTP7: *pka1Δ mal3Δ*, TTP26: *cgs1Δ mal3-GFP*, TTP20: *pka1Δ mal3-GFP*, TTP24: *cgs1Δ mal3-13Myc*, TTP22: *pka1Δ mal3-13Myc*, TTP104: *pka1Δ Ch16*, TTP70: *mal3Δ Ch16*, TTP110: *pka1Δ sad1-mRFP*, TTP113: *mal3Δ sad1-mRFP*, TTP76: *GFP-atb2 sad1-mRFP*, and TTP218: *pka1Δ GFP-atb2 sad1-mRFP*) were constructed by genetic crossing and selection with markers using standard yeast genetic techniques [31].

### Plasmid construction and induction of expression under the *nmt1* promoter

The oligonucleotide primers used for plasmid construction in this study are listed in S1 Table. To construct pGBKT7-Mal3, the oligonucleotide primers Mal3-BF and MAL3-SSR were used to amplify a 927 bp fragment containing the complete *mal3* protein coding sequence from *S*. *pombe* cDNA. The amplified *mal3* gene fragment was digested with *Bam*HI and *Sal*I and ligated into the corresponding sites of pGBKT7 (Clontech) to generate the plasmid pGBKT7-Mal3. To construct pGBKT7-Mal3 (1–143), pGBKT7-Mal3 (1–197), pGBKT7-Mal3 (1–218), pGBKT7-Mal3 (1–241), and pGBKT7-Mal3 (135–308), the pGBKT7-Mal3 plasmid was used as the template DNA. The oligonucleotide primer set MAL3-BF and MAL3-143SSmR for pGBKT7- Mal3 (1–143), the primer set MAL3-BF and MAL3-197SSmR for pGBKT7- Mal3 (1–197), the primer set MAL3-BF and MAL3-218SSmR for pGBKT7- Mal3 (1–218), the primer set MAL3-BF and MAL3-241SSmR for pGBKT7- Mal3 (1–241), and the primer set MAL3-135BF and MAL3-SSR for pGBKT7-Mal3 (135–308) were used to construct the corresponding plasmids. To construct pGAD424-Mal3, pGAD424-Mal3 (1–143), pGAD424-Mal3 (1–197), pGAD424-Mal3 (1–218), pGAD424-Mal3 (1–241), and pGAD424-Mal3 (135–308), each of the pGBKT7-derived plasmids was digested with *Bam*HI and *Sal*I and ligated into the corresponding sites of pGAD424 (Clontech).

To construct pREP3X-Mal3, pREP3X-Mal3 (1–143), pREP3X-Mal3 (1–197), pREP3X-Mal3 (1–218), pREP3X-Mal3 (1–241), and pREP3X-Mal3 (135–308), each of the pGBKT7-derived plasmids was digested with *Bam*HI and *Sma*I and cloned into the corresponding sites of pREP3X [32]. To construct pREP3X-Mal3 (Q89E) and pREP3X-Mal3 (Q89R), pGBKT7-Mal3 plasmid was used as the template DNA. The oligonucleotide primer sets MAL3-BF/MAL3(Q89E)-R and MAL3(Q89E)-F/MAL3-SSR were used to construct pREP3X-Mal3 (Q89E), and the primer sets MAL3-BF/MAL3(Q89R)-R and MAL3(Q89R)-F/MAL3-SSR were used to construct pREP3X-Mal3 (Q89R).

The plasmids pEGFP-N-human EB1 [33] and pGFP-NKB-mouse EB1 [34] were used as the template and the oligonucleotide primer sets HsMAPRE1-XF and HsMAPRE1-BR, and MmMAPRE1-XF and MmMAPRE1-BR were used to construct pREP3X-HsMAPRE1 (human EB1) and pREP3X-MmMAPRE1 (mouse EB1), respectively. The plasmids pET32-FHP-ATEB1a, pET32-FHP-ATEB1b, and pET32-FHP-ATEB1c [35] were used as the template and the oligonucleotide primer sets ATEB1a-SF and ATEB1a-BR, ATEB1b-SF and ATEB1b-BR, and ATEB1c-SF and ATEB1c-BR were used to construct pREP3X-AtEB1a (*Arabidopsis thaliana* EB1), pREP3X-AtEB1b (*A*. *thaliana* EB1b), and pREP3X-AtEB1c (*A*. *thaliana* EB1c), respectively. *Saccharomyces cerevisiae* genomic DNA was used as the template and the oligonucleotide primer set BIM1-SF and BIM1-BR was used to construct pREP3X-BIM1 (*S*. *cerevisiae* EB1).

To construct pGBKT7-Tip1, the oligonucleotide primers Tip1-BF and Tip1-SSR were used to amplify a 1,386 bp fragment containing the complete *tip1* protein coding sequence from *S*. *pombe* genomic DNA. The amplified *tip1* gene fragment was digested with *Bam*HI and *Sal*I and ligated into the corresponding sites of pGBKT7 to generate the plasmid pGBKT7-Tip1. To construct pGAD424-Tip1, the plasmid pGBKT7-Tip1 was digested with *Bam*HI and *Sal*I and ligated into the corresponding sites of pGAD424.

*S*. *pombe* genomic DNA from the Mal3-GFP strain (TTP3) was used as the template and the oligonucleotide primer set MAL3-BF and GFP-SR was used to construct pREP41X-Mal3-GFP. To construct pREP41X-Mal3(1–143)-GFP and pREP41X-Mal3(135–308)-GFP, the plasmids pREP41X-Mal3-GFP was used as the template and the oligonucleotide primer sets MAL3-BF, MAL3(1–143)GFP-F, MAL3(1–143)GFP-R, and GFP-SR for pREP41X-Mal3(1–143)-GFP, and MAL3-135BF and GFP-SR for pREP41X-Mal3(135–308)-GFP were used. The amplified fragments were digested with *Bam*HI and *Sma*I sites, and ligated into the appropriate sites of pREP41X.

The wild-type, *pka1Δ*, or *mal3Δ* cells were transformed with the pREP3X-derived plasmids (*LEU*2 marker) and selected onto EMMU (EMM containing uracil but lacking leucine) plates which also contained 15 μM thiamine to repress the gene expression under the *nmt1* promoter. To test the growth following *mal3* overexpression, transformants were grown on EMMU containing 15 μM thiamine for 2 days at 30°C, followed by transferred onto EMMU without thiamine to induce the *mal3* gene expression under the *nmt1* promoter, and incubated for 1 day at 30°C. The cells were then spotted on EMMU plates containing TBZ with or without 15 μM thiamine, and incubated for 3 to 5 days at 30°C.

When the expression of Mal3 was induced from the *nmt41* promoter, cells were first grown in EMM containing 15 μM thiamine, to the mid-log phase (~5 x 10^6^ cells/mL), at 30˚C. The cells were washed three times with water and resuspended in EMM lacking thiamine. The cells were incubated for 48 h at 30˚C.

### Mini-chromosomal loss assay

This assay was performed as previously described [36]. Two independent Ade^+^ isolates of the wild type, *pka1Δ*, and *mal3Δ* strains, were analyzed for the stability of the mini-chromosome16 (Ch16) containing the *ade6-M210* mutation, on adenine-limited EMMU plates, in the presence or absence of 7.5 μg/mL TBZ. The host *ade6-M210* cells are rendered Ade^+^ by allelic complementation. Transformed cells were plated on adenine-limited EMMU plates in the presence or absence of 7.5 μg/mL TBZ and incubated at 30˚C for 3 to 5 days and then at 4˚C for 1 to 2 overnight periods to allow deepening of the red color of the Ade^-^ colonies. The frequency of chromosome loss was determined by counting the total colonies and the red colonies.

### Yeast two-hybrid system

The yeast Matchmaker Two-Hybrid System 3 (Clontech) was used to examine the *in vivo* self-interaction of the truncated Mal3 proteins. For the assays, the plasmids of two fusion constructs into pGBKT7 (a Gal4 DNA-binding domain vector for Gal4-BD fusion) and pGAD424 (a Gal4-activating domain vector for Gal4-AD fusion) were then co-introduced into AH109 yeast cells using the lithium acetate method. Transformed yeast cells were grown on the synthetic dextrose medium (SC) lacking leucine and tryptophan (SC-LW) plate, for 3 days at 30˚C. The specific protein-protein interaction was determined by the growth of yeast cells on SC medium lacking leucine, tryptophan, and histidine (SC-LWH).

### Fluorescence microscopy of GFP fusion protein

*S*. *pombe* cells were grown in EMMU liquid medium containing 15 μM thiamine, washed three times with water, transferred to the EMMU liquid medium lacking thiamine, and incubated for 24 h at 30°C. The GFP-tagged Mal3 and the mRFP-tagged Sad1 proteins in living cells were visualized and imaged using a BX51 microscope (Olympus) with a DP70 digital camera (Olympus) or BZ-X700 microscope (Keyence).

### Preparation of cell lysates and detection of 13Myc fusion protein by immunoblotting

*S*. *pombe* cell lysates were prepared as previously described [37]. Protein lysates were separated by SDS-PAGE, after which western blot analysis was performed using an ECL detection system (GE Healthcare) according to the supplier’s instructions. Mouse monoclonal anti-Myc (diluted 1:1000) and rabbit polyclonal anti-PSTAIRE (Cdc2; diluted 1:1000) antibodies were purchased from Santa Cruz Biotechnology. Horseradish peroxidase-conjugated anti-mouse IgG (Santa Cruz Biotechnology) or anti-rabbit IgG antibody (Promega) was used as the secondary antibody.

### Data and statistical analyses

Experiments were performed three times and the average values and standard deviations (SD) were calculated. Data from the control and the overexpression of Mal3 were compared using the two-sample *t*-test. P-values < 0.05 were considered statistically significant. All statistical analyses were performed using EZR version 3.5.1 (Saitama Medical Center, Jichi Medical University, Saitama, Japan) [38], which is a graphical user interface for R (The R Foundation for Statistical Computing, Vienna, Austria).

### Reproducibility

All experiments were conducted at least twice to confirm the reproducibility of the results.

## Results

### Multi-copy Mal3 suppresses the TBZ- and MBC-sensitive phenotype of the *pka1Δ* strains

**Fig 1:**
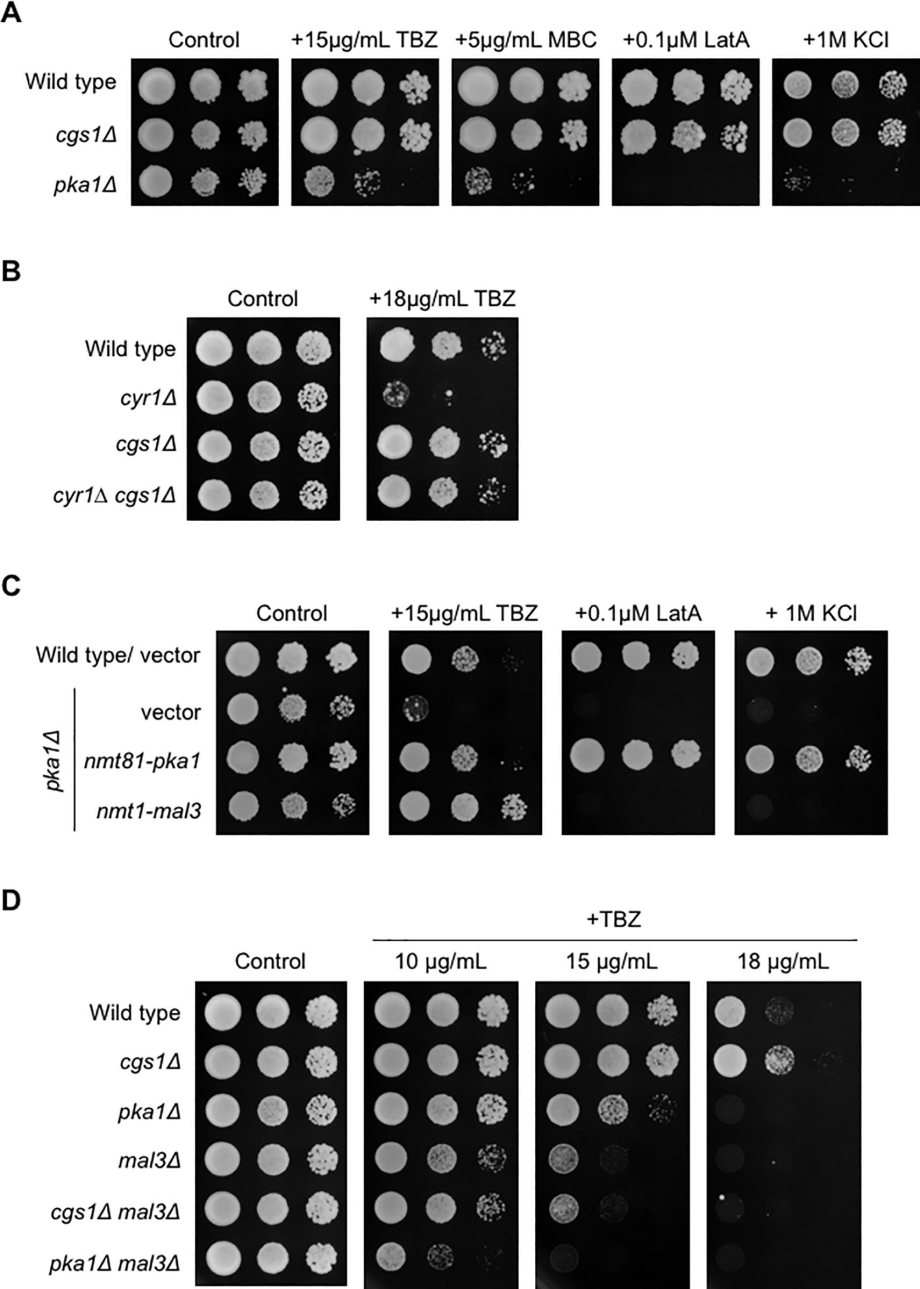
Overexpression of Mal3 suppresses the TBZ-sensitive phenotype of the *pka1Δ* strains. (A) Wild type (PR109), *cgs1Δ* (YMP40), and *pka1Δ* (YMP36) *S*. *pombe* strains were grown on YES, harvested, and resuspended in water at 10^7^ cells/mL. The cell suspensions were serially diluted (1:10) and each dilution was spotted onto YES in the presence or absence of 15 μg/mL TBZ, 5 μg/mL MBC, 0.1 μM LatA, 1 M KCl, and incubated for 5 days at 30°C. (B) Wild type (PR109), *cyr1Δ* (YMP28), *cgs1Δ* (YMP40), and *cyr1Δ cgs1Δ* (YMP36) *S*. *pombe* strains were grown on YES, harvested, and resuspended in water at 10^7^ cells/mL. Culture dilutions were prepared as described in Fig 1A and spotted on YES in the presence or absence of 18 μg/mL TBZ. The plates were incubated for 3 or 5 days at 30˚C (YES for 3 days and YES+18μg/mL TBZ for 5 days). (C) The wild type (PR109) and *pka1Δ* (YMP36) strains harboring pREP3X (vector), pREP81-pka1, or pREP3X-mal3 were cultured for 1 day on EMMU (EMM with uracil) containing 15 μM thiamine at 30°C to repress its expression of the *mal3* gene by the *nmt1* promoter. Cells were washed three times with water, transferred onto EMMU lacking thiamine and incubated for 24 h at 30°C to induce *mal3* by the *nmt1* promoter. Culture dilutions were prepared as described above and spotted onto EMMU in the presence or absence of 15 μg/mL TBZ, 0.1 μM LatA, or 1 M KCl. All plates were incubated for 5 days at 30˚C. (D) Wild type (PR109), *cgs1Δ* (YMP40), *pka1Δ* (YMP36), *mal3Δ* (TTP1), *cgs1Δ mal3Δ* (TTP5), and *pka1Δ mal3Δ* (TTP7) *S*. *pombe* strains were grown on YES, harvested, and resuspended in water at 10^7^ cells/mL. Culture dilutions were prepared as described in Fig 1A and spotted on YES in the presence of absence of 10 μg/mL TBZ, 15 μg/mL TBZ, or 18 μg/mL TBZ. All plates were incubated for 5 days at 30˚C.

To gain further the insight into the novel functions of Pka1 in *S*. *pombe*, the sensitivity of the wild type, *cgs1Δ*, and *pka1Δ* strains toward various drugs were tested. While the wild type and *cgs1Δ* strains grew on YES containing 0.1 μM Latrunculin A (LatA), 15 μg/mL thiabendazole (TBZ), 5 μg/mL carbendazim (MBC), or 1 M KCl, the *pka1Δ* strain did not on 0.1 μM LatA or 1 M KCl containing media as previously shown [20, 22, 39, 40]. We found the *pka1Δ* strain also exhibited growth retardation by 15 μg/mL TBZ and 5 μg/mL MBC (Fig 1A), which are microtubule-destabilization drugs. We next analyzed the role of Cyr1 and its dependency of Pka1 activity on the growth retardation phenotype by TBZ. We found that the *cyr1Δ* strain exhibited the TBZ-sensitive phenotype at the concentration of 18 μg/mL TBZ, as similarly observed in the *pka1Δ* strain, and deletion of *cgs1* reversed its phenotype (Fig 1B). Deletion of *cyr1* results in the inactivation of Pka1 and concomitant deletion of *cgs1* results in the constitutive activation of Pka1. These findings indicate that the TBZ-sensitive phenotype in the *cyr1Δ* strain is dependent on the Pka1 activity.

Because this phenotype of the *pka1Δ* strains was novel, we first analyzed mitotic microtubule of the *pka1Δ* strain. Both the wild type and *pka1Δ* strains showed normal mitotic microtubules structure in the presence of TBZ similar to its absence (S1 Fig). Because mitotic microtubules of the *pka1Δ* strain was indistinguishable with the wild type strain, we next conducted a screening for a multi-copy suppressor to identify the possible target of Pka1. The *pka1Δ* strain was transformed with pREP3X-*S*. *pombe* cDNA library, which is under the control of thiamine-repressible *nmt1* promoter, and screened on EMMU containing 15 μg/mL TBZ. As a result, we obtained 15 candidates including Mal3, which is a microtubule plus-end EB1 family protein. Next, we examined whether Mal3 is a specific multi-copy suppressor for the TBZ-sensitive phenotype of the *pka1Δ* strain. Toward this, the *pka1Δ* strain was transformed with pREP3X, pREP81-pka1, or pREP3X-mal3 and the transformed cells were spotted onto EMMU plates in the presence or absence of 15 μg/mL TBZ. Overexpression of Mal3 clearly rescued the TBZ-sensitive phenotype of the *pka1Δ* strains, although it did not rescue the growth on 1 M KCl or 0.1 μM LatA (Fig 1C). These results indicate that Mal3 is a specific multi-copy suppressor for TBZ sensitivity in the *pka1Δ* strain.

To exclude the possibility of Mal3 abnormality in the *pka1Δ* strains, we analyzed the expression and localization of Mal3 in the Mal3-13Myc *pka1Δ* and Mal3-GFP *pka1Δ* strains. As a result, the expression of Mal3-13Myc was not affected in the MBC treated *pka1Δ* strains (S2A Fig). Mal3-GFP was also normally localized at the microtubule in the *pka1Δ* strains after treatment with 20 μg/mL TBZ (S2B Fig). These results indicate that the function of Mal3 was not depressed in the *pka1Δ* strains. Because the *mal3Δ* strain exhibited the TBZ-sensitive phenotype similar to the *pka1Δ* strains [11, 41], we analyzed the genetic interaction between Pka1 and Mal3 by growing the *cgs1Δ mal3Δ* and *pka1Δ mal3Δ* double mutants on YES containing TBZ. As shown in Fig 1D, the wild type and *cgs1Δ* strains were not sensitive to TBZ, but the *cgs1Δ mal3Δ* double mutant as well as the *mal3Δ* strains exhibited sensitivity to 15 μg/mL TBZ. The TBZ sensitivity of the *pka1Δ mal3Δ* double mutant, however, was enhanced as it did not grow on a lower concentration of TBZ (10 μg/mL TBZ) compared with the *pka1Δ* or *mal3Δ* strains. The *cgs1Δ* strain also exhibited the TBZ-tolerance phenotype compared to the wild type strain (Fig 1D). The result, that the deletion of *pka1* enhances the TBZ sensitivity of the *mal3Δ* strain, suggests that Pka1 has other targets in addition to Mal3.

### Overexpression of the CH domain of Mal3 suppresses the TBZ-sensitive phenotype of the *pka1Δ* strain

**Fig 2:**
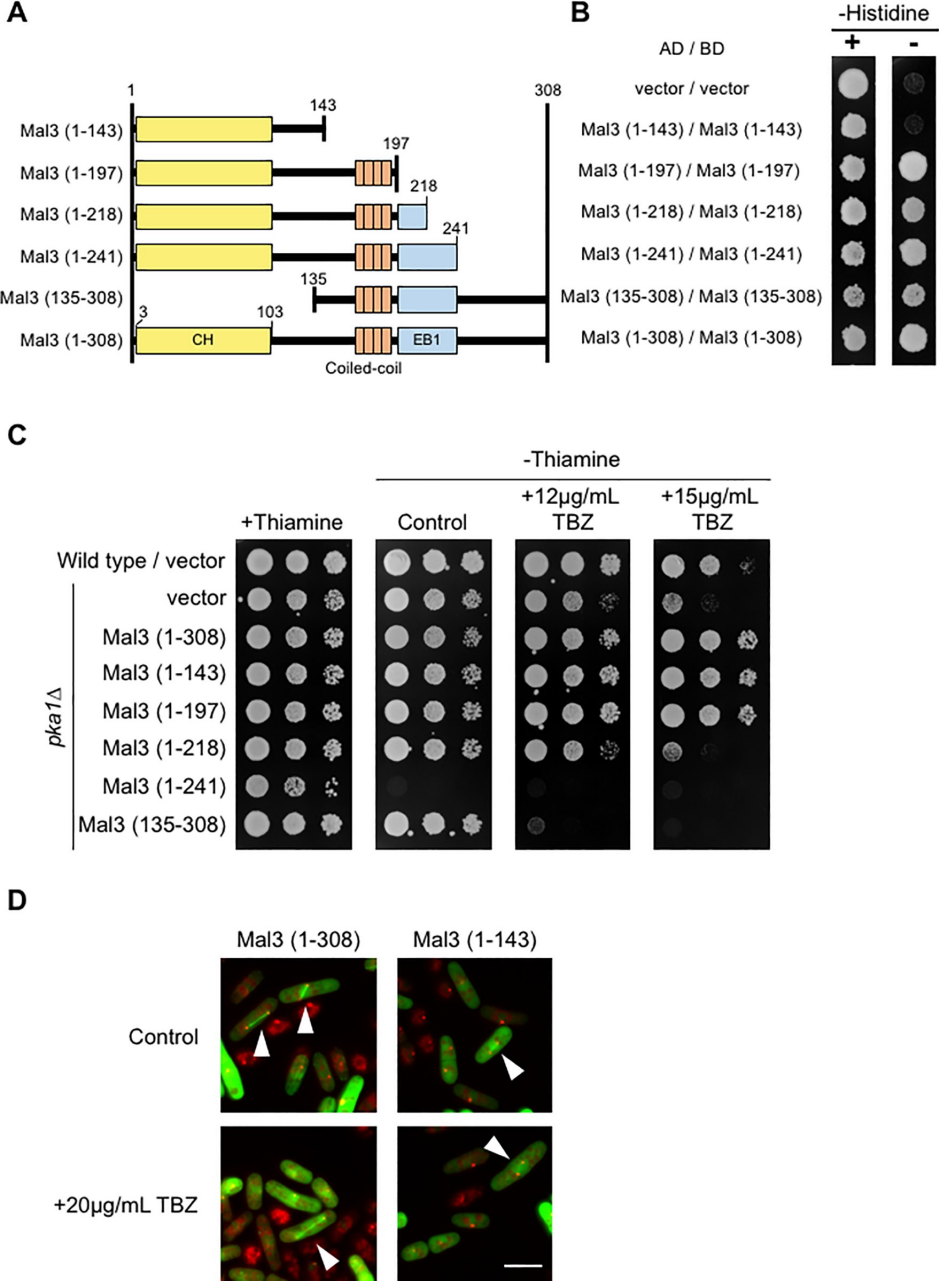
Interaction of individual domains of Mal3 and the suppression of the TBZ-sensitive phenotype in the *pka1Δ* strains by the CH domain. (A) The truncated Mal3 fragments used in this study. Mal3 has three domains; the CH domain (yellow), coiled-coil region (orange), and EB1 domain (blue). (B) *S*. *cerevisiae* AH109 strain was co-transformed with pGBKT7-derived plasmid and pGAD424-derived plasmid and selected on SC lacking leucine and tryptophan (SC-LW). Transformed cells were grown on SC-LW, harvested, and resuspended in water at 10^7^ cells/mL. The cell suspensions were adjusted to 0.4 at OD_600_, spotted onto SC-LW in the presence or absence of histidine (SC-LW or SC-LWH), and incubated for 4 days at 30˚C. (C) Wild type (PR109) and *pka1Δ* (YMP36) strains harboring pREP3X (vector), pREP3X-mal3 (1–308: full-length), pREP3X-mal3 (1–143), pREP3X-mal3 (1–197), pREP3X-mal3 (1–218), pREP3X-mal3 (1–241), or pREP3X-mal3 (135–308) were cultured as described in Fig 1C. Culture dilutions were prepared as described in Fig 1A and spotted onto EMMU in the presence or absence of 12 μg/mL TBZ or 15 μg/mL TBZ. (D) *pka1Δ* Sad1-mRFP (TTP110) strains harboring pREP41-Mal3 (1–308)-GFP or pREP41-Mal3(1–143)-GFP was grown in EMMU containing 15 μM thiamine for 1 day at 30˚C. Cells were washed three times by water, transferred into EMMU without thiamine, and incubated for 24 h at 30˚C. Mal3-GFP (green color) and Sad1-mRFP (red color) were observed by fluorescent microscopy; Sad1 localizes to spindle pole body which is similar to centrosome in mammals. Sad1-mRFP was used to visualize the end of microtubules. Arrowheads indicate mitotic microtubules. Scale bar: 10 μm.

Mal3 has three domains namely, the Calponin homology (CH) domain at the N-terminus (3–103), a coiled-coil domain at the middle region (167–194), and an end binding (EB1) domain at the C-terminus (197–241) [12]. To identify which domain is important for the suppression of the TBZ sensitivity of the *pka1Δ* strains, we constructed plasmids containing specific parts of Mal3 as shown in Fig 2A. We first analyzed the interaction of the Mal3 fragments by a yeast two-hybrid system. Mal3 (1–197), Mal3 (1–218), Mal3 (1–241), and Mal3 (135–308) interacted each other as well as with the full-length Mal3 (1–308) (Fig 2B). However, Mal3 (1–143) did not interact with itself as in Fig 2B and also as previously described [42].

Next, we analyzed the suppression of the TBZ sensitivity of the *pka1Δ* strains by the individual domains. As shown in Fig 2C, the overexpression of Mal3 (1–143) and Mal3 (1–197) rescued the TBZ-sensitive phenotype of the *pka1Δ* strains, while Mal3 (1–218) showed no effect. Mal3 (135–308) exhibited a growth defect in the *pka1Δ* strains at 12 μg/mL TBZ, and Mal3 (1–241) exhibited growth inhibition even in the absence of TBZ (Fig 2C). Overexpression of the Mal3 CH domain is sufficient for the suppression of the TBZ sensitivity of the *pka1Δ* strains and the coiled-coil domain has a negative effect on growth (Fig 2C). We then analyzed the localization of Mal3 (full-length, 1–308) and Mal3 (1–143) by GFP fusion expressed under the *nmt41* promoter in the *pka1Δ sad1-mRFP* strain, to visualize the end points of the microtubules. As shown in Fig 2D, the full-length of Mal3-GFP clearly localized to the microtubules in interphase and mitosis. We did not observe any localization of Mal3 (1–143)-GFP in interphase, but detected localization on microtubules during prophase to anaphase in mitosis (Fig 2D, arrowheads). These findings indicate that the microtubular localization of the CH domain of Mal3 is important, but its self-interaction is not required for the suppression of the TBZ sensitivity of the *pka1Δ* strains.

### Microtubule binding ability of Mal3 is required for TBZ suppression and EB1 proteins from other eukaryotes suppress the TBZ sensitivity of the *pka1Δ* strains

**Fig 3:**
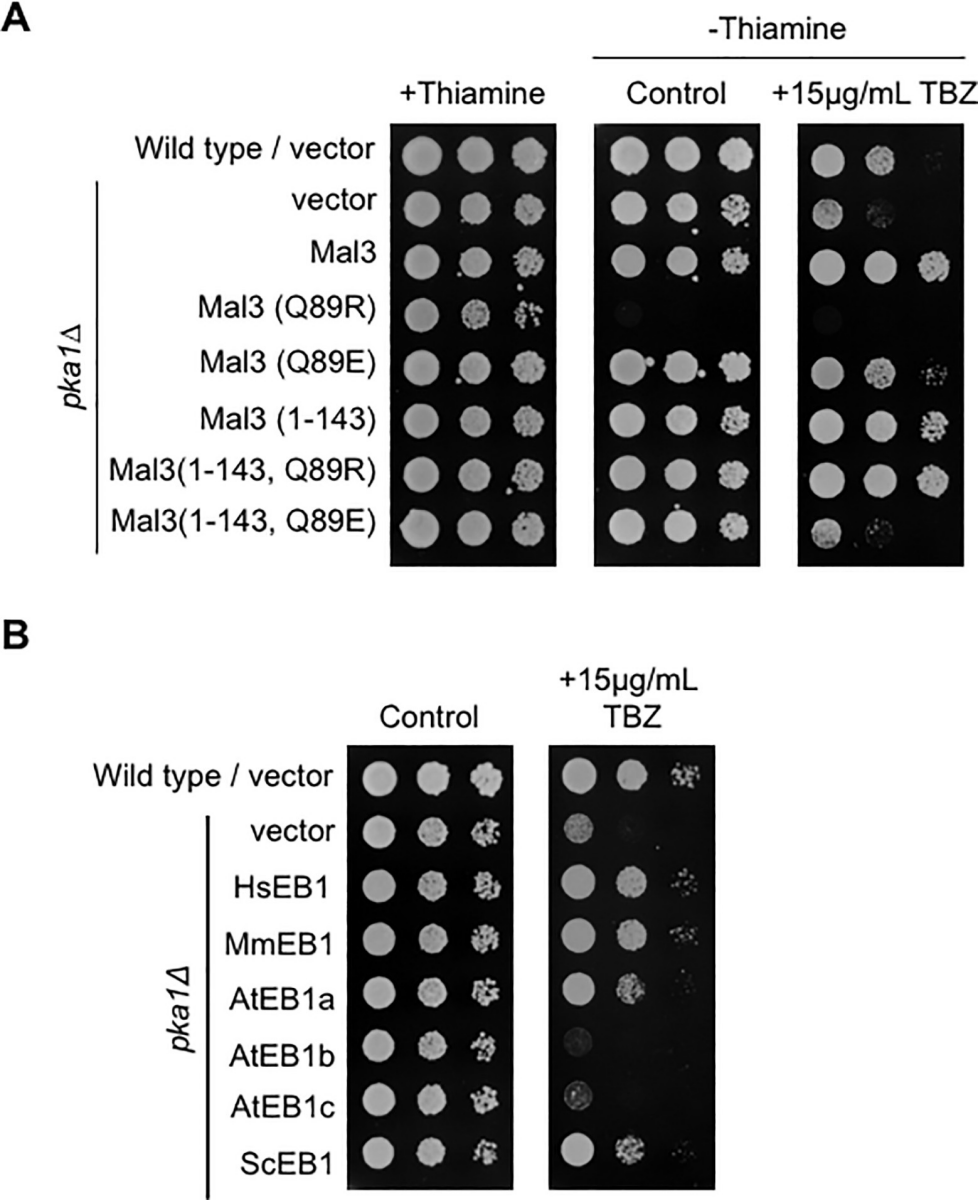
Microtubule binding of Mal3 is responsible for the suppression of the TBZ-sensitive phenotype in the *pka1Δ* strains. (A) Wild type (PR109) and *pka1Δ* (YMP36) strains harboring pREP3X (vector), pREP3X-mal3 (Q89R), pREP3X-mal3 (Q89E), pREP3X-mal3 (1–143), pREP3X-mal3 (1–143 Q89R), or pREP3X-mal3 (1–143 Q89E) were cultured as described in Fig 1C. Culture dilutions were prepared as described in Fig 1A and spotted on EMMU in the presence or absence of 15 μg/mL TBZ. All plates were incubated for 5 days at 30˚C. (B) Wild type (PR109) and *pka1Δ* (YMP36) strains harboring pREP3X (vector), pREP3X-HsMAPRE1 (HsEB1), pREP3X-MmMAPRE1 (MmEB1), pREP3X-AtEB1a (AtEB1a), pREP3X-AtEB1b (AtEB1b), pREP3X-AtEB1c (AtEB1c), or pREP3X-BIM1 (ScEB1) were cultured as described in Fig 1C. Culture dilutions were prepared as described in Fig 1A and spotted on EMMU in the presence or absence of 15 μg/mL TBZ. All plates were incubated for 5 days at 30˚C.

It has been previously reported that the Mal3 (Q89R) mutant strongly binds to the microtubules, and the Mal3 (Q89E) mutant does not [12, 13]. We used the Mal3 mutants (Q89R and Q89E) to identify whether microtubule binding of Mal3 is required for the suppression TBZ sensitivity of the *pka1Δ* strains on EMM. As shown in Fig 3A and Table 2, overexpression of the full-length Mal3 (Q89R) mutant caused growth inhibition in the *pka1Δ* strains in the absence of thiamine (control panel) and the full-length Mal3 (Q89E) weakly rescued the growth of the *pka1Δ* strains on 15 μg/mL TBZ. To further clarify the suppression by the Mal3 mutants, we next constructed the plasmids expressing the mutants of the CH domain: pREP3X-Mal3 (1–143 Q89E) and pREP3X-Mal3 (1–143 Q89R). Mal3 (1–143 Q89R) resulted in the rescue of the *pka1Δ* strains on EMM in the presence of 15 μg/mL TBZ or 5 μg/mL MBC, while Mal3 (1–143 Q89E) showed no effect on the *pka1Δ* strains on the same media (Fig 3A and Table 2). These results indicate that the microtubule binding of Mal3 CH domain is important for the suppression of sensitivity to the microtubule-destabilizing drugs, in the *pka1Δ* strains.

**Table 2:** Growth profile of the *pka1Δ* strains upon overexpression of various Mal3 domains or orthologs.

Plasmid	No drug	+15 μg/mL TBZ	+5 μg/mL MBC	+10 μg/mL MBC
vector	+	-	-	-
Mal3 (1–308)	+	+	+	+
Mal3 (1–143)	+	+	+	-
Mal3 (1–197)	+	+	+	-
Mal3 (1–218)	+	-	-	-
Mal3 (1–241)	-	-	-	-
Mal3 (135–308)	+	-	-	-
Mal3 (Q89E)	+	+	+	-
Mal3 (Q89R)	-	-	-	-
Mal3 (1–143 Q89E)	+	-	-	-
Mal3 (1–143 Q89R)	+	+	+	-
HsEB1	+	+	+	-
MmEB1	+	+	+	-
AtEB1a	+	+	+	-
AtEB1b	+	-	-	-
AtEB1c	+	-	-	-
ScEB1	+	+	+	-
Tip1	+	-	N. T.	N. T.
Tea1	+	-	N. T.	N. T.
Tea2	-	-	N. T.	N. T.
Alp7	+	-	N. T.	N. T.
Alp14	+	-	N. T.	N. T.

The TBZ sensitivity and the MBC sensitivity were analyzed by spotting assay and streak assay, respectively. N.T.: not tested.

Mal3 localizes to the microtubule plus end as a plus-end tracking protein (+TIP), thereby controlling cell morphology and microtubule dynamics in fission yeast. Therefore, we next examined whether the other +TIP proteins such as Tip1 (CLIP-170), Tea1 (cell end marker), and Tea2 (kinesin), exhibit the suppression of the TBZ-sensitive phenotype of the *pka1Δ* strains. As a result, the overexpression of Tip1, Tea1, or Tea2 did not rescue the growth defect of the *pka1Δ* strains on EMM containing 15 μg/mL TBZ, although the overexpression of Tea2 caused growth defect under normal condition (S3 Fig and Table 2). We also examined the effect of the other microtubule binding proteins Alp7 (TACC protein) and Alp14 (TOG/XMAP215). As a result, Alp7 or Alp14 did not rescue the TBZ-sensitive phenotype of the *pka1Δ* strain (S3 Fig and Table 2). These results indicate that the CH domain of Mal3 has a specific role in the suppression of sensitivity to the microtubule-destabilizing drugs of the *pka1Δ* strains.

Next, we addressed whether the suppressive effect of Mal3 is conserved in higher eukaryotic EB1. To address this, we examined the effect of human EB1 (HsEB1), mouse EB1 (MmEB1), *A*. *thaliana* EB1 (AtEB1a, AtEB1b, and AtEB1c), and *S*. *cerevisiae* Bim1p (ScEB1). As shown in Fig 3B and Table 2, the overexpression of HsEB1, MmEB1, AtEB1a, and ScEB1 rescued the TBZ-sensitive or MBC-sensitive phenotype of the *pka1Δ* strains. However, the overexpression of AtEB1b and AtEB1c showed no effects on the growth of the *pka1Δ* strains on EMM with 15 μg/mL TBZ or 5 μg/mL MBC (Fig 3B and Table 2). Therefore, the higher eukaryotic Mal3 orthologs as well as the budding yeast Mal3 have conserved function in regard to the suppression of TBZ-sensitive phenotype of the *pka1Δ* strains.

### Overexpression of Mal3 (1–143) suppresses the chromosome mis-segregation induced by TBZ in the *pka1Δ* strains

**Fig 4:**
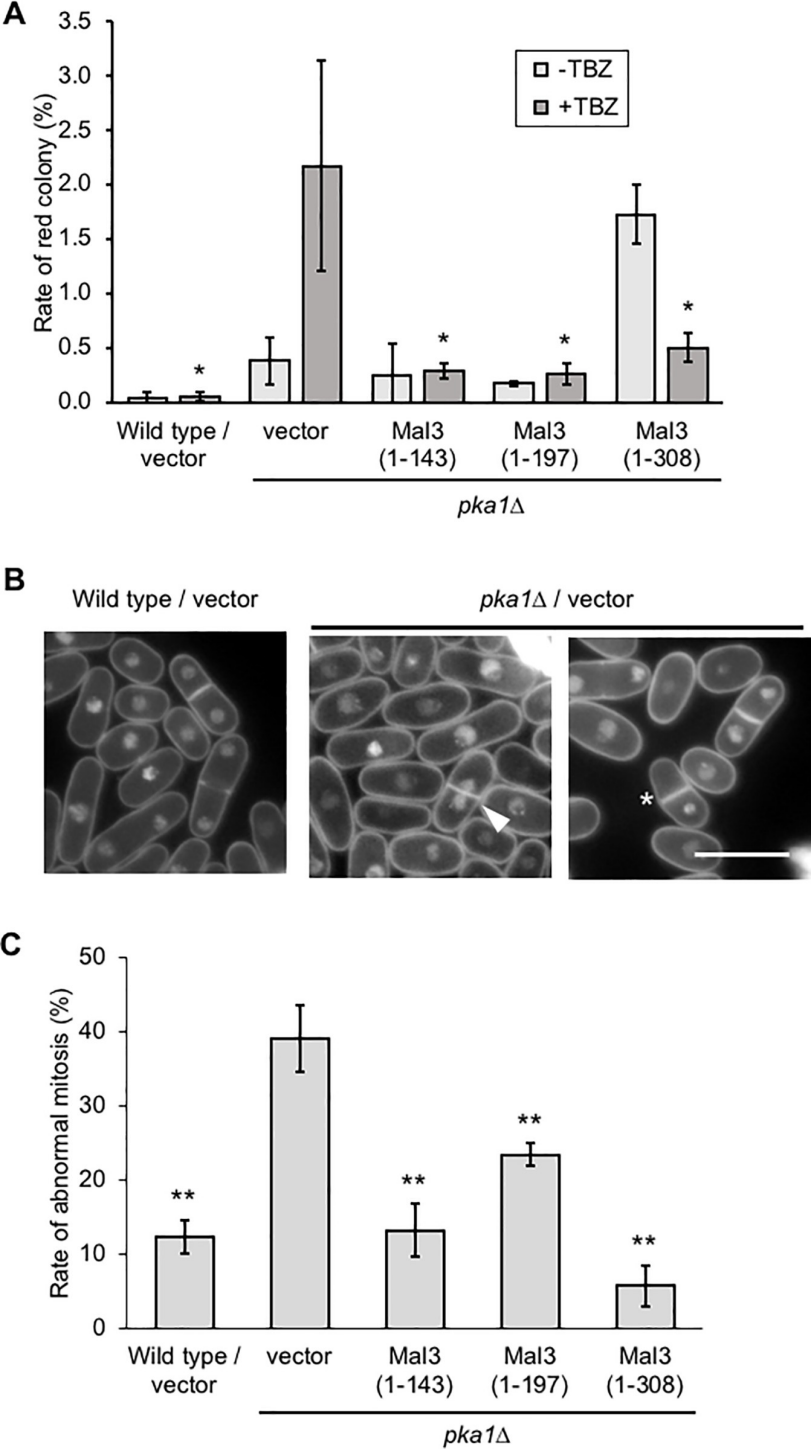
Overexpression of Mal3 (1–143) suppresses chromosomal mis-segregation in the *pka1Δ* strains caused by the microtubule-destabilizing drugs. (A) The wild type (TTP101) and *pka1Δ* (TTP104) strains harboring mini-chromosome Ch16 were transformed with pREP3X (vector), pREP3X-mal3 (1–143), pREP3X-mal3 (1–197), or pREP3X-mal3 (1–308; full-length). Cells were cultured as described in Fig 1C. Cultured cells were platted on EMMU containing 10 μg/mL adenine in the presence or absence of 7.5 μg/mL TBZ. Plates were incubated for 7 days at 30˚C. Red colonies and sector colonies were counted. Experiments were performed three times: averages with S.D. are shown. Asterisks (*) indicate P-value < 0.05 for comparison with the *pka1Δ* strain harboring pREP3X (vector). (B) Wild type (PR109) and *pka1Δ* (YMP36) strains were cultured in YES. Cells were incubated with 10 μg/mL TBZ for 24 h at 18˚C. Cells were stained with DAPI and observed by fluorescent microscopy. Arrowhead and asterisk show the *cut* phenotype and mis-segregation, respectively. Scale bar: 10 μm. (C) Abnormal mitosis including the *cut* phenotype, in which cytokinesis takes place over the unseparated chromosomes and mis-segregation was determined by DAPI staining. About 50 cells were analyzed in individual strains and experiments were performed three times; averages with S.D. are shown. Double asterisks (**) indicate P-value < 0.01 for comparison with the *pka1Δ* strain harboring pREP3X (vector).

The observation that the Mal3 (1–143) rescues the TBZ-sensitive phenotype of the *pka1Δ* strains, and localizes to mitotic microtubules, led us to hypothesize that Mal3 (1–143) may suppress the chromosome mis-segregation of the *pka1Δ* strains. To address this, we analyzed the chromosome segregation by the mini-chromosome loss assay. Because mini-chromosome Ch16 has the *ade6* gene and in the strains that have the *ade6-M210* mutation, the colonies show white color when the strains normally segregate the mini-chromosome, whereas the colonies show red color when the strains mis-segregated the mini-chromosome, on a plate containing low concentration of adenine [36]. Wild type strain harboring the vector exhibited white color colonies in the presence of 7.5 μg/mL TBZ, whereas the *pka1Δ* strain harboring the vector yielded approximately 0.5% red colonies in the absence of TBZ and approximately 2% red colonies in the presence of 7.5 μg/mL TBZ (Fig 4A). Overexpression of the Mal3 (full-length), Mal3 (1–143), or Mal3 (1–197) rescued the mini-chromosome loss of the *pka1Δ* strain in the presence of TBZ (Fig 4A). Overexpression of Mal3 (135–308) inhibited the growth on low concentration of TBZ (5 μg/mL) and exhibited a high frequency of red colonies on EMMU in the absence of TBZ (data not shown). Next, we analyzed the chromosome mis-segregation by DAPI staining using fluorescence microscopy. The wild type strain showed approximately 10% chromosome mis-segregation in the presence of 10 μg/mL TBZ, whereas the *pka1Δ* strains exhibited a high frequency (approximately 40%) of abnormal mitosis including the *cut* phenotype in which cells are separated by the septum before nuclear division and mis-segregated nucleus (Fig 4B and 4C, and Table 3). Mal3 (full-length; 1–308), Mal3 (1–143), and Mal3 (1–197) overexpressing cells rescued the abnormal mitosis phenotype in the *pka1Δ* strains in the presence of 10 μg/mL TBZ (Fig 4C and Table 3), indicating that the CH domain of Mal3 rescued the abnormal chromosome segregation in the *pka1Δ* strains.

**Table 3:** The ratio of abnormal mitosis in the presence of 10 μg/mL TBZ.

Strain	Plasmid	Mis-segregation (%)	Cut phenotype (%)	Total (%)
Wild type	vector	8.5 +/- 0.04	3.9 +/- 0.02	12.4 +/- 2.3
*pka1Δ*	vector	29.6 +/- 0.06	9.5 +/- 0.04	39.1 +/- 4.5
	Mal3 (1–143)	8.8 +/- 0.03	4.5 +/- 0.03	13.3 +/- 3.5
	Mal3 (1–197)	18.8 +/- 0.01	4.7 +/- 0.01	23.5 +/- 1.6
	Mal3 (1–308)	4.9 +/- 0.02	0.8 +/- 0.01	5.7 +/- 2.8

### Overexpression of the Mal3 CH domain suppresses the growth defect and chromosome mis-segregation in the presence of TBZ in the *mal3Δ* strains

**Fig 5:**
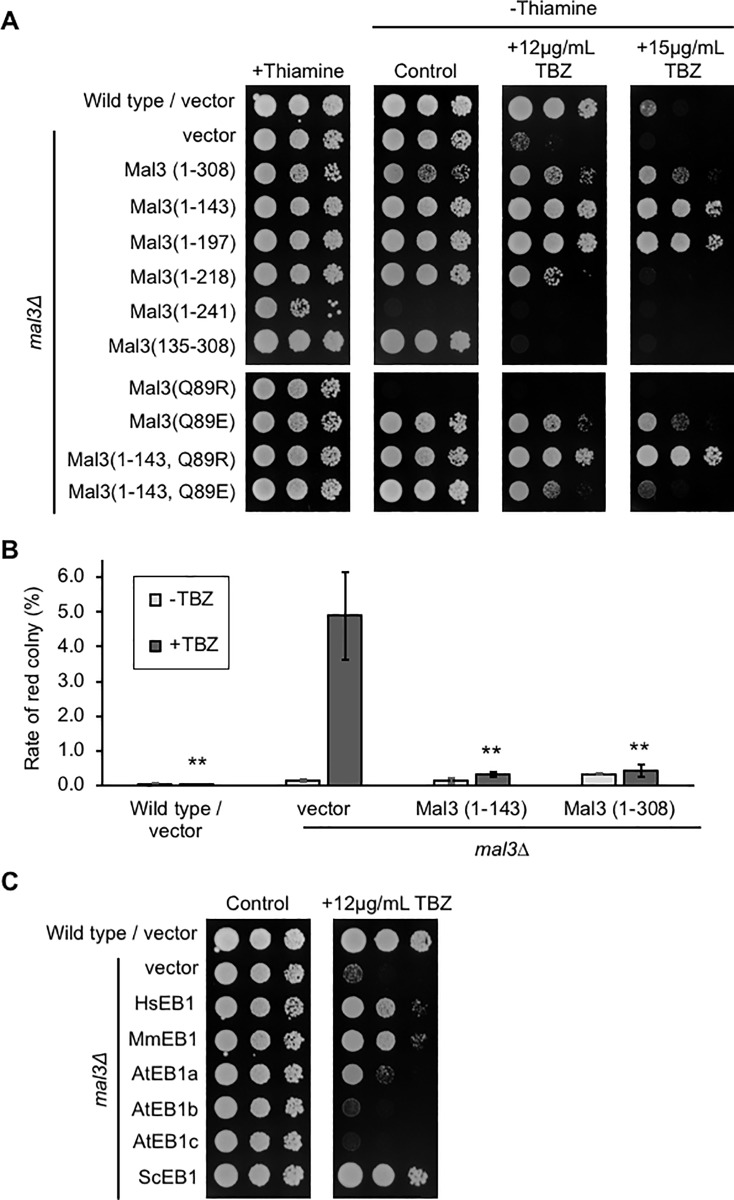
Overexpression of Mal3 (1–143) suppresses the growth defect and chromosome mis-segregation caused by TBZ in the *mal3Δ* strains. (A) Wild type (PR109) and *mal3Δ* (TTP1) strains harboring pREP3X (vector), pREP3X-mal3 (1–143), pREP3X-mal3 (1–308; full-length), pREP3X-mal3 (1–197), pREP3X-mal3 (1–218), pREP3X-mal3 (1–241), pREP3X-mal3 (135–308), pREP3X-mal3 (Q89R), pREP3X-mal3 (Q89E), pREP3X-mal3 (1–143 Q89R), or pREP3X-mal3 (1–143 Q89E) were cultured as described in Fig 1C. Culture dilutions were prepared as described in Fig 1A and spotted on EMMU in the presence or absence of 12 μg/mL TBZ or 15 μg/mL TBZ. All plates were incubated for 5 days at 30˚C. (B) Wild type (TTP69) and *mal3Δ* (TTP70) strains harboring the mini-chromosome Ch16 were transformed with pREP41X (vector), pREP41X-mal3 (1–143), or pREP41X-mal3 (1–308; full-length). Cells were cultured as described in Fig 1C. Cultured cells were platted on EMMU containing 10 μg/mL adenine in the presence or absence of 7.5 μg/mL TBZ. Plates were incubated for 7 days at 30˚C. Red colonies and sector colonies were counted. Experiments were performed three times; averages with S.D. are shown. Double asterisks (**) indicate P-value < 0.01 for comparison with the *mal3Δ* strain harboring pREP3X (vector). (C) Wild type (PR109) and *mal3Δ* (TTP1) strains harboring pREP3X (vector), pREP3X-mal3, pREP3X-HsMAPRE1 (HsEB1), pREP3X-MmMAPRE1 (MmEB1), pREP3X-AtEB1a (AtEB1a), pREP3X-AtEB1b (AtEB1b), pREP3X-AtEB1c (AtEB1c), or pREP3X-BIM1 (ScEB1) were cultured as described in Fig 1C. Culture dilutions were prepared as described in Fig 1A and spotted on EMMU in the presence or absence of 12 μg/mL TBZ. All plates were incubated for 5 days at 30˚C.

It has been previously reported that *mal3Δ* results in the TBZ-sensitive phenotype and chromosome mis-segregation [11]. We next analyzed whether the deletion of the CH domain causes the TBZ-sensitive phenotype in the *mal3Δ* strains. Toward this, we used the plasmids expressing the fragmented and mutated Mal3. The overexpression of Mal3 (1–143), Mal3 (1–197), Mal3 (1–218), and Mal3 (1–308 full-length) rescued the growth of the *mal3Δ* strains on EMM in the presence of 15 μg/mL TBZ, whereas the overexpression of Mal3 (135–308) exhibited growth retardation on 12 μg/mL TBZ (Fig 5A and Table 4). The overexpression of Mal3 (1–241) exhibited growth inhibition even in the absence of TBZ as observed in the *pka1Δ* strain (Fig 5A and Table 4). Full-length Mal3 (Q89E) and Mal3 (Q89R) exhibited growth suppression on EMM containing TBZ and growth inhibition on EMM in the *mal3Δ* strain, respectively. Because these results might be due to the effect of the EB1 domain, we next analyzed using the fragmented mutants of Mal3: Mal3 (1–143 Q89R) and Mal3 (1–143 Q89E). Overexpression of Mal3 (1–143 Q89R), which is an enhanced microtubule binding mutant, rescued the growth defect of the *mal3Δ* strains, similar to that observed in the *pka1Δ* strains, on EMM containing 15 μg/mL TBZ (Fig 5A and Table 4) or 10 μg/mL MBC (Table 4). The mutant defective in microtubule binding, Mal3 (1–143 Q89E), did not restore the sensitivity to 15 μg/mL TBZ (Fig 5A and Table 4) or 5 μg/mL MBC in the *mal3Δ* strains, similar to that observed in the *pka1Δ* strains (Table 4), indicating that the microtubule binding of Mal3 CH domain is required for the growth in the presence of microtubule-destabilizing drug.

**Table 4:** Growth profile of the *mal3Δ* strains upon overexpression of various Mal3 domains or orthologs.

Plasmid	No drug	+15 μg/mL TBZ	+5 μg/mL MBC	+10 μg/mL MBC
vector	+	-	-	-
Mal3 (1–308)	+	+	+	+
Mal3 (1–143)	+	+	+	+
Mal3 (1–197)	+	+	+	+
Mal3 (1–218)	+	+	+	-
Mal3 (1–241)	-	-	-	-
Mal3 (135–308)	+	-	-	-
Mal3 (Q89E)	+	+	+	-
Mal3 (Q89R)	-	-	-	-
Mal3 (1–143 Q89E)	+	-	-	-
Mal3 (1–143 Q89R)	+	+	+	+
HsEB1	+	+	+	-
MmEB1	+	+	+	-
AtEB1a	+	+	+	-
AtEB1b	+	-	-	-
AtEB1c	+	-	-	-
ScEB1	+	+	+	-

The TBZ sensitivity and the MBC sensitivity were analyzed by spotting assay and streak assay, respectively.

Next, we analyzed whether the CH domain is functional for proper chromosome segregation in the *mal3Δ* strains, by the mini-chromosome loss assay. As results, the overexpression of the Mal3 CH domain (1–143) or the Mal3 full-length (1–308) rescued the mini-chromosome loss in the *mal3Δ* strains, similar to that observed in the *pka1Δ* strains (Fig 5B). Whereas, the overexpression of the Mal3 EB1 domain (135–308) inhibited the growth in the presence of TBZ (data not shown). These results indicate that the CH domain of Mal3 plays a role of maintenance in the normal segregation of chromosome.

Next, we analyzed the suppression upon overexpression of other eukaryotic EB1s in the *mal3Δ* strains. Overexpression of *S*. *cerevisiae* Bim1p (ScEB1), human EB1 (HsEB1), mouse EB1 (MmEB1), and *A*. *thaliana* EB1a (AtEB1a) rescued the growth defect in the *mal3Δ* strains in the presence of 12 μg/mL TBZ but *A*. *thaliana* EB1b (AtEB1b) and EB1c (AtEB1c) failed to do so, as observed in the *pka1Δ* strains (Fig 5C). These results indicate that the EB1 from yeasts to higher eukaryotes have conserved role in the restoration of growth defect in the presence of microtubule-destabilizing drugs.

### Overexpression of the Mal3 CH domain give rise to the TBZ-tolerance in the wild type strain

**Fig 6:**
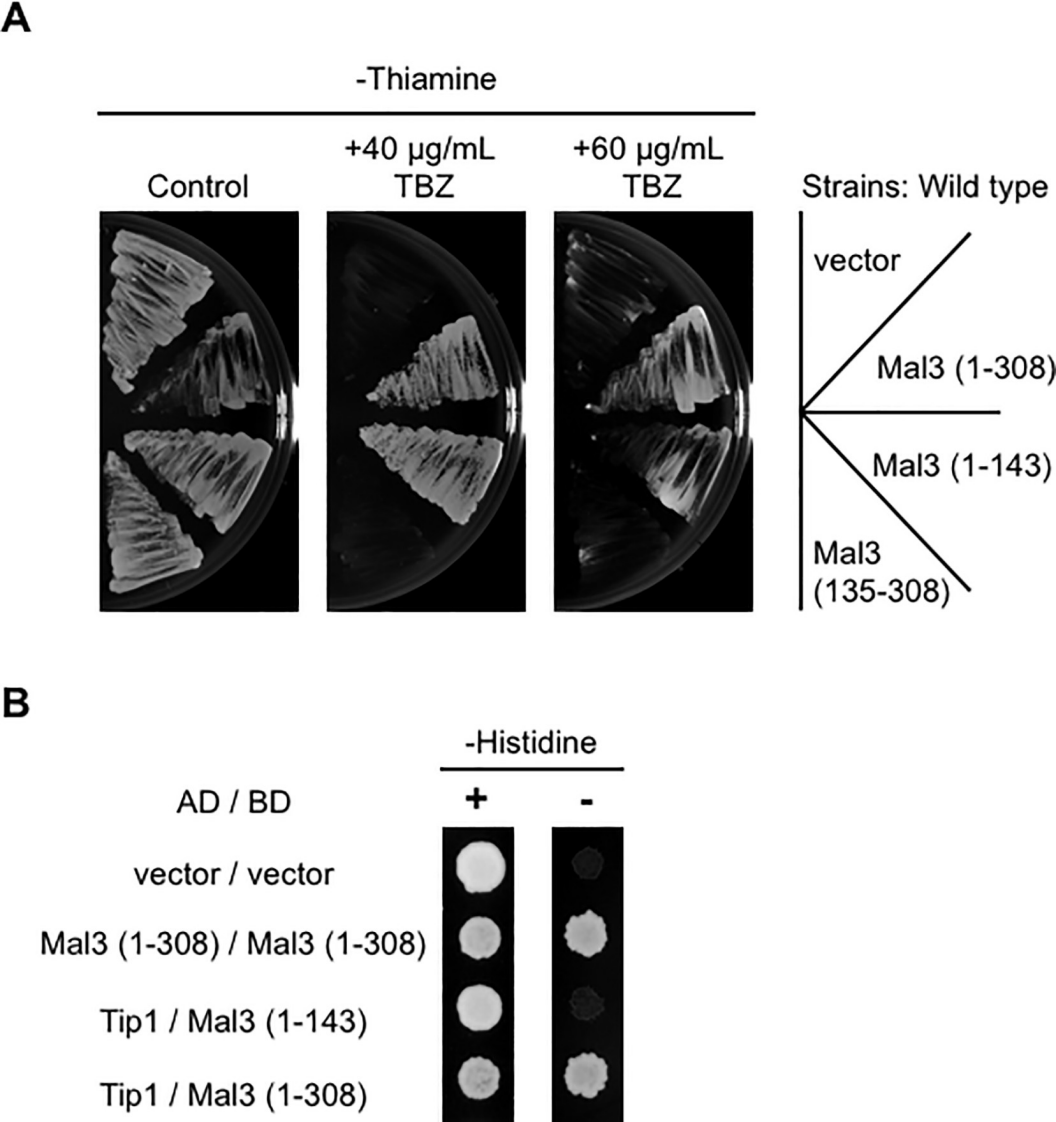
Overexpression of Mal3 (1–143) exhibits the TBZ-tolerance in the wild type strain. (A) Wild type (PR109) strains harboring pREP3X (vector), pREP3X-mal3 (1–308; full-length), pREP3X-mal3 (1–143), or pREP3X-mal3 (135–308) were cultured as described in Fig 1C. Cells were streaked on EMMU in the presence or absence of 40 μg/mL TBZ or 60 μg/mL TBZ. All plates were incubated for 5 days at 30˚C. (B) *S*. *cerevisiae* AH109 strain was co-transformed with pGBKT7-derived plasmid and pGAD424-derived plasmid and selected on SC lacking leucine and tryptophan (SC-LW). Transformed cells were cultured and the cell suppression was spotted as described in Fig 2B. All plates were incubated for 3 days at 30˚C.

As we observed that overexpression of Mal3 (1–143) rescued the TBZ-sensitive phenotypes of *pka1Δ* and *mal3Δ* strains, we analyzed the effect of Mal3 (1–143)-overexpression in wild type by looking at its phenotype. The wild type strain was transformed with pREP3x, pREP3x-mal3 (1–308; full-length), pREP3X-mal3 (1–143), or pREP3X-mal3 (135–308). Transformants were selected on EMMU in the presence of 15μM thiamine. To test the TBZ- sensitivity, transformants were cultured on EMMU in the absence of thiamine, transferred on EMMU in the presence or absence of TBZ, and incubated at 30 ˚C. As a result, overexpression of Mal3 (1–308) or Mal3 (1–143) did not alter their growth even with the presence of a high concentration of TBZ (60 μg/mL), although Mal3 (135–308)-overexpression caused inhibition of cell growth (Fig 6A). This result suggests that the *pka1Δ* and *mal3Δ* strains were rescued by the TBZ-tolerance of the Mal3 CH domain (1–143).

Finally, we tested the possibility that the Mal3 CH domain would affect the CLIP170 ortholog, Tip1. To do this, we analyze the interaction between the Mal3 CH domain and Tip1 by the yeast two-hybrid system. As a result, Mal3 (1–143) did not interact with Tip1 although Mal3 (1–308: full-length) interacted with Tip1 (Fig 6B). This result suggests that the suppression of the Mal3 CH domain was not mediated by Tip1.

## Discussion

It has been reported that Pka1 is involved in glucose repression, chronological aging, regulation of transit from mitosis to meiosis, and stress response to KCl and CaCl_2_ in *S*. *pombe* [14, 20–25]. Pka1 also regulates the transition from the G2 phase to M phase, as the overexpression of Pka1 also causes growth defect and mitotic defects leading to cell elongation and DNA content accumulation [43]. On the other hand, PKA regulates the transition of G1/S phase in *S*. *cerevisiae* and the progression of G2/M phase in *Xenopus laevis* [44, 45]. These reports suggest that PKA regulates cell cycle progression at several phases. However, its detailed mechanism is mostly unclear. In the M phase, *S*. *pombe* Pka1 is involved in the regulation of the anaphase-promoting complex (APC), but its detailed mechanism has not been elucidated [46–50]. In this study, we found that *pka1Δ* exhibits the TBZ-sensitive phenotype and abnormal chromosome segregation in the presence of TBZ. In general, microtubule-destabilizing drugs inhibit the progression of the M phase in the cell cycle. Spindle assembly checkpoint (SAC) proteins such as Mad1, Mad2, Mad3, Bub1, Bub3, and Mph1 are not essential for the growth under normal condition. However, inactivated SAC causes the TBZ-sensitive phenotype and chromosome mis-segregation in the presence of microtubule destabilization drug [51]. SAC proteins negatively regulate APC for progression from metaphase to anaphase in the M phase. These results and our observation, that the *pka1Δ* strains exhibit the TBZ-sensitive phenotype and chromosome mis-segregation, together suggest that Pka1 regulates SAC and/or APC in the M phase.

To know how Pka1 regulates chromosome segregation, we performed multi-copy suppressor screening and isolated Mal3 (MAPs and +TIP protein) based on its ability to suppress TBZ sensitivity in the *pka1Δ* strains. The CH domain (1–143) of Mal3, but not other domains, is responsible for this suppression. Our results suggest that the deletion of the *pka1* gene prevents the attachment of chromosome to the microtubule and the CH domain of Mal3 enhances the binding stability between the microtubule and chromosome. In fact, a microtubule unattached form of Mal3 (1–143 Q89E) failed to restore the sensitive of TBZ in the *pka1Δ* strains (Fig 3A). The loss of functional Dis1, a TOG/XMAP215 microtubule plus end tracking polymerase, resulted in chromosome mis-segregation [52]. Leucine 841 and proline 844 of Dis1 at C-terminus are responsible for binding to Mal3, while the C-terminus region from 174 to 247 amino acids residue containing a part of coiled-coil domain and EB1 domain of Mal3 are required for binding with Dis1 [41]. Based on their report, we suggest that the function of Dis1 is not required for the suppression of the TBZ-sensitive phenotype in the *pka1Δ* strain. The CH domain of Mal3 (1–143) did not localize to the microtubules during interphase and only localized to the microtubules during prophase to anaphase. The +TIP proteins, Tea1, Tea2, and Tip1, mostly play a role of in the cell polarity of microtubules in interphase [53]. Tea1, Tea2, and Tip1 bind through Mal3 to the microtubules [53]. This is because the formation of +TIP complex mediated by EB1 domain of Mal3 is required to attach to microtubules in interphase, and the CH domain alone is not able to play this role as observed in the case of Tea1, Tea2, and Tip1 [53, 54]. Therefore, the function of mitotic microtubules is important for suppression of the TBZ-sensitive phenotype in the *pka1Δ* strain. Overexpression of Alp14, which is a microtubule binding protein containing the TOG domain, functions similar to the CH domain of Mal3, but failed to restore the TBZ sensitive phenotype in the *pka1Δ*, indicating that the CH domain of Mal3 is required for microtubule stability in the *pka1Δ* strains. The CH domain of Mal3 does not show discernible differences from the TOG domain regarding the Pka1-mediated microtubule stabilization.

Beinhauer *et al*. have shown that the *mal3Δ* strain exhibited the TBZ-sensitive phenotype and chromosome mis-segregation [11]. These results were also supported in this study (Figs 1D and 5). The CH domain of Mal3 (1–143) is sufficient to suppress these phenotypes, while an unbinding form of Mal3 (1–143 Q89E) failed to reverse the TBZ sensitive phenotype of the *mal3Δ* strains (Fig 5 and Table 4). These results indicate that the microtubule binding ability of Mal3 is important for proper function. It has been reported that the CH domain of Mal3 binds to microtubules *in vitro* [13], but function of the CH domain has not been clearly understood *in vivo*. Our result in this study showed that the CH domain of Mal3 co-localizes with the microtubules and retains a role required for the proper segregation of chromosome. It has also been shown that the Mal3 (Q89R) mutant strongly binds to the microtubules and causes hyper phosphorylation at serine and threonine between 144 and 155 amino acids [12]. Overexpression of Mal3 (full-length Q89R) exhibited sever growth defect even under normal growth condition, probably due to excessive binding to microtubule; whereas overexpression of Mal3 (full-length Q89E) showed suppressive effect on the TBZ-sensitive phenotype of the *pka1Δ* and *mal3Δ* strains; even the mutant has only weak microtubule binding ability, probably due to the moderate effect of this mutation. Intriguingly, the effect of Q89 mutation was different when a truncated version of Mal3 was expressed. While Mal3 (1–143 Q89R) suppressed the TBZ-sensitive phenotype of the *pka1Δ* and *mal3Δ* strains, Mal3 (1–143 Q89E) failed to do so. This suggests that Mal3 (1–143 Q89E) does not have sufficient microtubule binding ability comparing with Mal3 (full-length Q89E) when overexpressed. We also observed that the overexpression of Mal3 (1–241) causes sever growth inhibition compared to full-length Mal3 and other Mal3 fragmented proteins (Figs 2C and 5A, and our unpublished data). These results suggest that the growth inhibition is caused by overexpression of fragments, including both the CH domain and the EB1 domain. The analysis to understand the cause of the severe growth inhibition observed with the truncated C-terminus mutant is currently underway. Genetic analysis on the *pka1Δ* and *mal3Δ* mutants suggest that these two genes work in parallel pathway, because the *pka1Δ mal3Δ* double mutant exhibited much severe sensitivity to TBZ than each single mutant (Fig 1D). Overexpression of Pka1 did not rescue the TBZ-sensitive phenotype of *mal3Δ* (data not shown), supporting the idea that Mal3 operates downstream of the cAMP/PKA pathway during mitosis. In fact, the gain of functional PKA mutant, the *cgs1Δ* strain, rescued the TBZ sensitivity in the *cyr1Δ* strain (Fig 1B) and exhibited the TBZ-tolerance phenotype compared to the wild type strain (Fig 1D). This result suggests that the PKA activity is important for the response to the microtubule-destabilizing drugs.

Finally, we showed that the functional suppression is also observed in the EB1 orthologs *S*. *cerevisiae* Bim1p (ScEB1), human EB1 (HsEB1), mouse EB1 (MmEB1), and *A*. *thaliana* EB1a (AtEB1a), in the *pka1Δ* and *mal3Δ* strains. EB1 protein has the CH domain at the N-terminus and EB1 domain at the C-terminus. In all these organisms, the EB1 protein binds to the microtubules and plays the role of microtubule stabilization [55]. We identified that the EB1 proteins is responsible for proper chromosome segregation by genetic analysis. Our complementation analysis indicates that the function of Mal3 is mostly conserved from yeasts to high eukaryotes, as the overexpression of AtEB1b and AtEB1c did not suppress the TBZ-sensitivity of the *pka1Δ* and *mal3Δ* strains (Fig 3B). Because AtEB1c has a tail region with patches of basic amino acid residues, its function and structure are different from Mal3, budding yeast Bim1p (ScEB1), mouse EB1 (MmEB1), human EB1 (HsEB1), and AtEB1a [35]. Our suppression results suggest that the function is conserved in fission yeast Mal3, budding yeast Bim1p (ScEB1), mouse EB1 (MmEB1), human EB1 (HsEB1), and *A*. *thaliana* AtEB1a. However, we have no explanation on why the overexpression of AtEB1b failed to suppress the TBZ-sensitive phenotype in the *pka1Δ* and *mal3Δ* strains.

In conclusion, in addition to the known roles of Pka1 in meiosis, gluconeogenesis, chorological aging, and stress response of *S*. *pombe*, we propose that Pka1 is also involved in the regulation of chromosome segregation during mitosis. Our findings provide new insights into the novel function of Pka1 in regard to the CH domain of Mal3, in the regulation of microtubule organization and chromosome segregation in *S*. *pombe*.

## Supporting information

S1 Fig The *pka1Δ* strain shows normal mitotic microtubules. *GFP-atb2 sad1-mRFP* (TTP76) and *pka1Δ GFP-atb2 sad1-mRFP* (TTP218) strains were cultured in EMMLU (EMM+leucine+uracil) liquid medium to mid-log phase (~4 × 10^6^ cells/mL). Cells were cultured for 30 min in EMMLU liquid medium in the presence or absence of 20 μg/mL TBZ. Cells were observed by fluorescence microscopy. Green and red colors show GFP-Atb2 and Sad1-mRFP, respectively. Scale bar: 10 μm.
(TIFF)

S2 Fig Expression and localization of Mal3 are not changed in the *cgs1Δ* and *pka1Δ* strains. (A) *mal3-13Myc* (TTP4), *cgs1Δ mal3-13Myc* (TTP24), and *pka1Δ mal3-13Myc* (TTP22) strains were cultured in YES liquid medium to mid-log phase (~4 × 10^6^ cells/mL), and after addition of 10 mM HU, the cells were incubated for 4 h to arrest in the S phase. Cells were harvested by centrifugation and resuspended in YES with 50 μg/mL MBC, and further incubated for 2 h to prepare the cell lysates. To prepare asynchronous cells (As), the cells were cultured in YES liquid medium to mid-log phase (~4 × 10^6^ cells/mL). Mal3-13Myc protein were detected by an anti-Myc antibody. Anti-PSTAIRE was used as an internal loading control. (B) *mal3-GFP* (TTP3), *cgs1Δ mal3-GFP* (TTP26), and *pka1Δ mal3-GFP* (TTP20) strains were cultured in YES liquid medium to mid-log phase (~4 × 10^6^ cells/mL), and after addition of 10 mM HU, the cells were incubated for 4 h to arrest in the S phase. Cells were harvested by centrifugation and resuspended in YES with 20 μg/mL TBZ. Cells were observed by fluorescence microscopy at 2 h after incubation with TBZ. Scale bar: 10 μm.
(TIFF)

S3 Fig Overexpression of +TIP proteins expect Mal3 failed to restore the TBZ-sensitive phenotype in the *pka1Δ* strains. (A) Wild type (PR109) and *pka1Δ* (YMP36) strains harboring pREP3X (vector), pREP3X-tip1, pREP3X-tea1, pREP3X-tea2, pREP3X-mal3, pREP41X-alp14, or pREP41X-alp7 were cultured as described in Fig 1C. Culture dilutions were prepared as described in Fig 1A and spotted on EMMU in the presence or absence of 18 μg/mL TBZ. All plates were incubated for 5 days at 30˚C.
(TIFF)

S1 Table Oligonucleotide primers used for making plasmids in this study. (DOCX)
