# Supplementary figures and images for: Mal3 is a multi-copy suppressor of the sensitivity to microtubule-depolymerizing drugs and chromosome mis-segregation in a fission yeast pka1 mutant
Source: PLoS One. 2019 Apr 11;14(4):e0214803. doi: 10.1371/journal.pone.0214803 (PMC6459531; doi:10.1371/journal.pone.0214803)

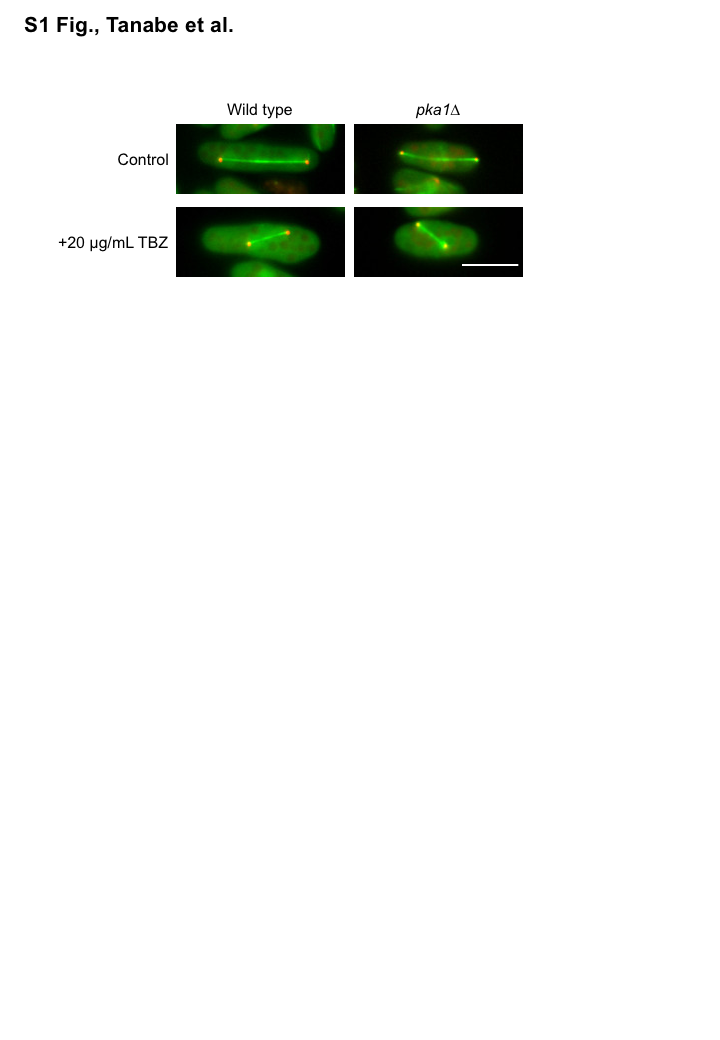

Supplement: S1 Fig — GFP-atb2 sad1-mRFP (TTP76) and pka1Δ GFP-atb2 sad1-mRFP (TTP218) strains were cultured in EMMLU (EMM+leucine+uracil) liquid medium to mid-log phase (~4 × 10^6^ cells/mL). Cells were cultured for 30 min in EMMLU liquid medium in the presence or absence of 20 μg/mL TBZ. Cells were observed by fluorescence microscopy. Green and red colors show GFP-Atb2 and Sad1-mRFP, respectively. Scale bar: 10 μm. (TIFF) [file pone.0214803.s001.tiff]

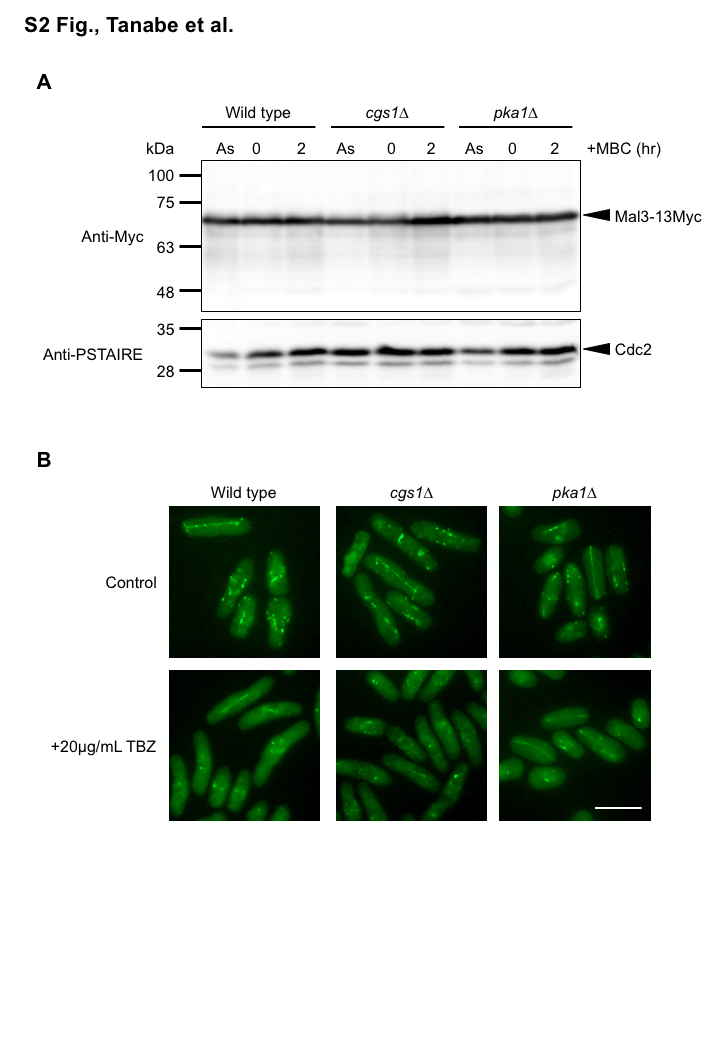

Supplement: S2 Fig — (A) mal3-13Myc (TTP4), cgs1Δ mal3-13Myc (TTP24), and pka1Δ mal3-13Myc (TTP22) strains were cultured in YES liquid medium to mid-log phase (~4 × 10^6^ cells/mL), and after addition of 10 mM HU, the cells were incubated for 4 h to arrest in the S phase. Cells were harvested by centrifugation and resuspended in YES with 50 μg/mL MBC, and further incubated for 2 h to prepare the cell lysates. To prepare asynchronous cells (As), the cells were cultured in YES liquid medium to mid-log phase (~4 × 10^6^ cells/mL). Mal3-13Myc protein were detected by an anti-Myc antibody. Anti-PSTAIRE was used as an internal loading control. (B) mal3-GFP (TTP3), cgs1Δ mal3-GFP (TTP26), and pka1Δ mal3-GFP (TTP20) strains were cultured in YES liquid medium to mid-log phase (~4 × 10^6^ cells/mL), and after addition of 10 mM HU, the cells were incubated for 4 h to arrest in the S phase. Cells were harvested by centrifugation and resuspended in YES with 20 μg/mL TBZ. Cells were observed by fluorescence microscopy at 2 h after incubation with TBZ. Scale bar: 10 μm. (TIFF) [file pone.0214803.s002.tiff]

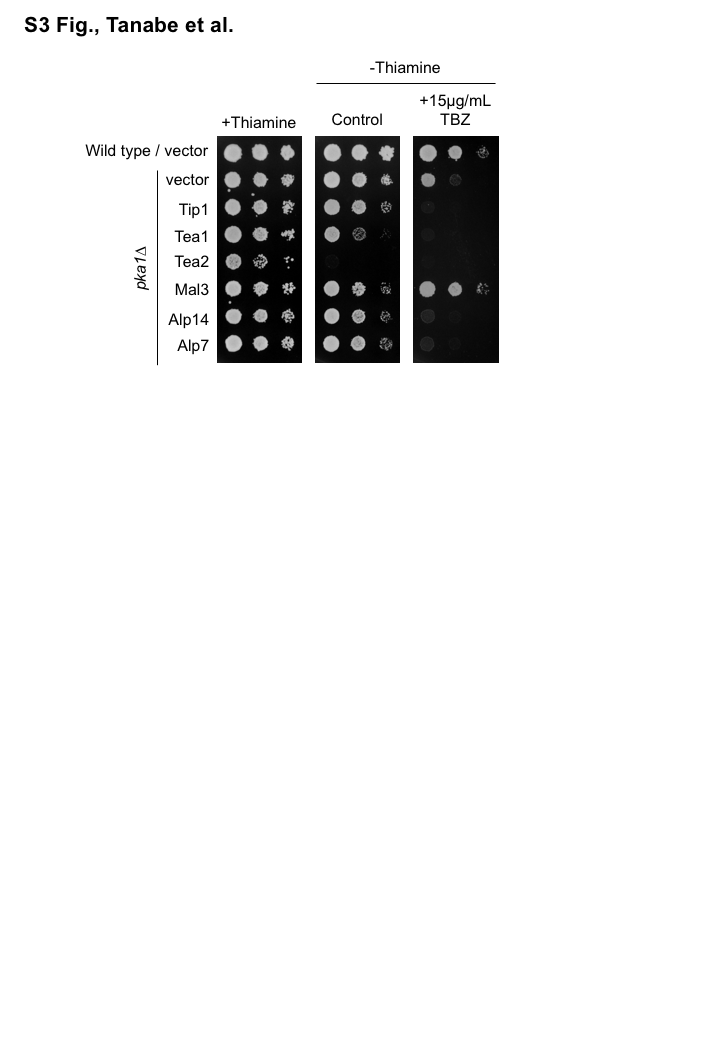

Supplement: S3 Fig — (A) Wild type (PR109) and pka1Δ (YMP36) strains harboring pREP3X (vector), pREP3X-tip1, pREP3X-tea1, pREP3X-tea2, pREP3X-mal3, pREP41X-alp14, or pREP41X-alp7 were cultured as described in Fig 1C. Culture dilutions were prepared as described in Fig 1A and spotted on EMMU in the presence or absence of 18 μg/mL TBZ. All plates were incubated for 5 days at 30˚C. (TIFF) [file pone.0214803.s003.tiff]
