# Supplementary material for: Mal3 is a multi-copy suppressor of the sensitivity to microtubule-depolymerizing drugs and chromosome mis-segregation in a fission yeast pka1 mutant
Source: PLoS One. 2019 Apr 11;14(4):e0214803. doi: 10.1371/journal.pone.0214803 (PMC6459531; doi:10.1371/journal.pone.0214803)
Supplement: S1 Table — (DOCX) [file pone.0214803.s004.docx]

**S1 Table Oligonucleotide primers used for making plasmids in this study.**

| Primer name | Sequence |
| --- | --- |
| MAL3-BF | 5’-TATGGATCCTCATGTCTGAATCTCGGCAAGAG-3’ |
| MAL3-SSR | 5’-ACAGTCGACCCGGGTTAAAACGTGATATTCTCATC-3’ |
| MAL3-135BF | 5’-TATGGATCCTCATGACTGGCCCTTCTCGTCGCCGTC-3’ |
| MAL3-143SSmR | 5’- ACAGTCGACCCGGGTTAAACCTGACGGCGACGAGAAG-3’ |
| MAL3-197SSmR | 5’-ACAGTCGACCCGGGTTAACCAAACATCGTCTCATTAAC-3’ |
| MAL3-218SSmR | 5’-ACAGTCGACCCGGGTTAAGTTTGTACAAGTATTTCAAT-3’ |
| MAL3-241SSmR | 5’-ACAGTCGACCCGGGTTAAGTAGAATAAAGTATTGCTTG-3’ |
| MAL3(Q89E)-F | 5’-TTGTAAAATGGAAGATAATCT-3’ |
| MAL3(Q89E)-R | 5’-AGATTATCTTCCATTTTACAA-3’ |
| MAL3(Q89R)-F | 5’-TTGTAAAATGAGAGATAATCTG-3’ |
| MAL3(Q89R)-R | 5’-CAGATTATCTCTCATTTTACAA-3’ |
| HsMAPRE1-XF | 5’-TATCTCGAGATGGCAGTGAACGTATACTC-3’ |
| HsMAPRE1-BR | 5’-ACAGGATCCTTAATACTCTTCTTGCTCCTC-3’ |
| MmMAPRE1-XF | 5’-TATCTCGAGATGGCAGTGAATGTGTACTC-3’ |
| MmMAPRE1-SmR | 5’-ACACCCGGGTTAATACTCTTCTTGTTCCTC-3’ |
| ATEB1a-SF | 5’-TATGTCGACATGGCGACGAACATCGGAATG-3’ |
| ATEB1a-BR | 5’-ACAGGATCCTTAGGCTTGAGTCTTTTCTTC-3’ |
| ATEB1b-SF | 5’-TATGTCGACATGGCGACGAACATTGGGATG-3’ |
| ATEB1b-BR | 5’-ACAGGATCCTTAAGTTTGGGTCTCTGCAG-3’ |
| ATEB1c-SF | 5’-TATGTCGACATGGCTACGAACATTGGGATG-3’ |
| ATEB1c-BR | 5’-ACAGGATCCTCAGCAGGTCAAGAGAGGAG-3’ |
| BIM1-SF | 5’-TATGTCGACATGAGTGCGGGTATCGGAGAATC-3’ |
| BIM1-BR | 5’-ACAGGATCCTTAAAAAGTTTCCTCGTCGATG-3’ |
| MAL3(1-143)GFP-F | 5’-CTTCTCGTCGCCGTCAGGTTTCGTACGCTGCAGGTCGACG-3’ |
| MAL3(1-143)GFP-R | 5’-CGTCGACCTGCAGCGTACGAAACCTGACGGCGACGAGAAG-3’ |
| GFP-SR | 5’-ACACCCGGGAGATCTATATTACCCTGTTA-3’ |
| TIP1-BF | 5’-TATGGATCCAAATGTTTCCTCTTGGCAGTGTC-3’ |
| TIP1-SSR | 5’-ACAGTCGACCCGGGTTAAGCTTCGTCTGTGCTG-3’ |
